# Sustainable Carbon Dots Loaded into Carboxymethylcellulose Based Hydrogels for Uterine Cancer Bioimaging

**DOI:** 10.3390/pharmaceutics16121500

**Published:** 2024-11-22

**Authors:** Jordane S. Rodrigues, Pedro Brandão, Sofia O. D. Duarte, Izabela Boueri da Silveira, Maria de Fátima Leite, Max P. Gonçalves, Fernanda G. L. Medeiros Borsagli, Pedro Fonte

**Affiliations:** 1 Institute of Engineering, Science and Technology, Universidade Federal dos Vales do Jequitinhonha e Mucuri/UFVJM, Av. 01, 4050 Cidade Universitária, Janaúba 39440-039, MG, Brazil; jordane.rodrigues@ufvjm.edu.br (J.S.R.);; 2 iBB-Institute for Bioengineering and Biosciences, Department of Bioengineering, Instituto Superior Técnico, University of Lisboa, 1049-001 Lisbon, Portugal; pbrandao@egasmoniz.edu.pt (P.B.); sofia.duarte@tecnico.ulisboa.pt (S.O.D.D.); 3 Associate Laboratory i4HB–Institute for Health and Bio-Economy, Instituto Superior Técnico, University of Lisboa, Av. Rovisco Pais, 1049-001 Lisbon, Portugal; 4 Egas Moniz Center for Interdisciplinary Research (CiiEM), Egas Moniz School of Health & Science, 2829-511 Almada, Portugal; 5 CQC-IMS, Department of Chemistry, University of Coimbra, Rua Larga, 3004-535 Coimbra, Portugal; 6 Department of Physiology and Biophysics, Institute of Biology Science, Universidade Federal de Minas Gerais/UFMG, Av. Antônio Carlos, 6627, Belo Horizonte 30130-100, MG, Brazilleitemd@ufmg.br (M.d.F.L.); 7 Center for Marine Sciences (CCMAR), University of Algarve, Gambelas Campus, 8005-139 Faro, Portugal; 8 Department of Chemistry and Pharmacy, Faculty of Sciences and Technology, University of Algarve, Gambelas Campus, 8005-139 Faro, Portugal

**Keywords:** carbon dots, uterine cancer, hydrogels, bioimaging, circular economy, biomaterials

## Abstract

**Background/Objectives**: The development of innovative materials for disease diagnostics and therapeutics is a fast-growing area of scientific research. In this work, we report the development of innovative hydrogels incorporating carbon dots (Cdots) for bioimaging purposes. **Methods**: The Cdots were prepared using a sustainable and low-cost process, starting with an underused fiber from the Brazilian semiarid region. Spectroscopy analysis (Fourier transform infrared spectroscopy, X-ray photoelectron spectroscopy, UV-visible spectroscopy), X-ray diffraction, photoluminescence, zeta potential, scanning electron microscopy, and transmission electron microscopy were used to characterize these hydrogels. In addition, biocompatibility using the resazurin assay and cellular uptake by confocal microscopy were evaluated. **Results**: Our results showed that the Cdots changed the structure and crystallinity of hydrogels, mainly due to heat treatment. In addition, hydrogels’ chemical groups suffer red and blue shifts following the Cdots incorporation. Moreover, the Cdots were homogeneously incorporated into the hydrogel matrix. Importantly, the cytotoxicity levels were maintained above 90% (*p* < 0.01), and cellular uptake studies using HeLa cells demonstrated intracellular fluorescence of both the Cdots and hydrogels after incubation. Additionally, the concentration of Cdots within hydrogels significantly affected fluorescence intensity, even compared with pure Cdots. **Conclusions**: These results showcase the potential for these hydrogels to be further developed as biomarkers and therapeutic biomaterials for women’s health.

## 1. Introduction

Uterine cancer is a significant global health concern, ranking as the sixth most common cancer among women worldwide. In 2020, there were approximately 417,367 new cases. It is also a leading cause of cancer-related deaths in women, accounting for about 97,370 fatalities. The lack of early diagnosis due to nonspecific symptoms and the absence of routine screening programs results in a higher proportion of advanced-stage diagnoses, impacting treatment outcomes. There is an urgent need to develop sustainable, cost-effective materials derived from biodegradable and biocompatible sources for uterine cancer diagnostics [1,2,3].

This delayed diagnosis significantly impacts treatment outcomes and overall survival rates (Brazilian Health Minister, 2016). The lack of dedicated screening initiatives targeting the detection of uterine cancer, as it happens for breast or cervical cancer, presents an added hurdle in the early detection of the disease [1,2]. Additionally, the search for non-invasive treatments is a great strategy that has been implemented in biomaterials for the diagnosis and treatment of several diseases, such as diabetes [4,5,6] and cancer.

In this context, in recent years, major advances in medicine, biology, pharmacology, and engineering have opened up promising avenues for women’s health, with the development of several innovative materials for treating, diagnosing, mapping, and treating various diseases. The latest advances in nanomedicine, an imminent sector of research integrating nanoscience, pharmaceutical technology, and medicine, facilitate the development of new diagnostic tools for early-stage cancer detection [7,8]. Moreover, strategies like the use of bioactive molecules [9], protein-loaded nanoparticles [10], nanoparticles with antioxidant reagents [11], and others have a high potential for clinical use in several diseases. Therefore, materials science faces a significant challenge in developing sustainable materials that are multifunctional, cost-effective, and robust while also being socially and economically viable [12,13].

In this context, the use of natural raw materials has become promising due to their biodegradability, renewable origins, simple manufacturing methods, and potential sourcing from waste across various sectors. Furthermore, the United Nations (UN) has advocated for sustainable global development approaches. In this regard, key objectives include poverty eradication, zero hunger, and sustainable agriculture, particularly relevant to impoverished regions and countries. These goals set by the UN foster a keen interest in materials that combine sustainability with enhanced mechanical properties and high performance. However, it is crucial to emphasize that developing these materials requires precise, meticulous, and thorough characterization protocols [14].

In this regard, carbon-based nanocrystals, such as carbon quantum dots or carbon dots (Cdots), present an interesting option as ultrasmall nanomaterials. They can be efficiently produced through chemical reactions, exhibit biocompatibility, are often derived from natural or agro-industrial sources, and are cost-effective [15,16]. These attributes align well with the sustainable development goals proposed by the UN. These nanocrystals have been studied as an impressive class of nanomaterials for bioimaging, as they possess excellent optical and electronic features [8]. Although several efforts were made in order to develop novel bioimaging methodologies to improve therapeutic outcomes, most of these techniques still present several constraints and limitations [16]. However, most Cdots production techniques are expensive and require high energy [17], and therefore, there is still room for improvement in sustainable Cdots production.

In essence, in a poor Brazilian semiarid region, advancing the two sustainable development goals proposed by the UN—particularly emphasizing reusability and the utilization of environmental resources for economic enhancement—is crucial. Notably, this region is home to the *Ceiba speciosa* tree, locally known as Paineira, which produces a substantial amount of fiber in spring for seed dispersal. Historically, these fibers have seen limited social or economic use [18,19]. The *Ceiba speciosa*, part of the Malvaceae family, plays a vital role in ecosystem restoration and has a wide geographic distribution in the Brazilian semiarid region [18,19], an area facing significant social and economic challenges exacerbated by severe droughts [20]. Thus, harnessing this natural resource to develop advanced nanomaterials for medical applications could contribute significantly to eradicating poverty and hunger while promoting sustainable agriculture in the area, thereby benefiting local economic and social development.

The fibers of *Ceiba speciosa* are characterized by a hollow cylindrical structure with a large lumen, distinguishing them from other fibers with a high porosity of nearly 80%, contributing to their lightness [20]. These fibers contain a substantial amount of cellulose along with a significant wax content, giving them a hydrophobic character and high cellulosic potential for the production of various carbon nanomaterials, such as carbon dots [20]. Moreover, using these fibers can enhance economic opportunities in economically disadvantaged regions of Brazil.

Then, the present work reports the synthesis of Cdots via a new and low-cost chemical route using *Ceiba speciosa* fibers and incorporated these Cdots into Carboxymethylcellulose hydrogels to evaluate the potential of these materials for bioimaging, namely uterine cancer. These hydrogels were thoroughly characterized using different techniques. Then, biological assays, including cellular uptake with confocal microscopy, were performed to determine their biocompatibility and biomarker potential.

## 2. Materials and Methods

### 2.1. Materials

Sodium salt of carboxymethyl cellulose (≥99.5%, Synth, Brazil, DS = 0.7, Mw = 250,000 g.mol^−1^), citric acid (C_6_H_8_O_7_, ≥99.5%, Synth, Brazil), sodium hydroxide (Neon, Brazil, ≥99%, NaOH), (CH_3_)_2_CHOH), acetic acid (Synth, Brazil, 99.8%, MM = 60.05 g.mol^−1^, CH_3_CO_2_H), hydrochloric acid (Neon, Brazil, 36.5–38.0%, HCl), sulfuric acid (Synth, Brazil, 99.8%, H_2_SO_4_), and sodium chloride (Synth, Brazil, 99.9%, NaCl) were used in this study. Deionized water (DI water) (Millipore Simplicity™, Merck, Rahway, NJ, USA) with a resistivity of 18 MΩ·cm was used. Fibers from *Ceiba speciosa* (a typical plant of semiarid/Brazilian *cerrado*) were used as feedstock to produce the Cdots. The fibers of *Ceiba speciosa* collected corresponded to the “seed hair” that allows the seeds to spread in the Brazilian spring. The fibers were collected in the north region of Minas Gerais (semiarid region), Brazil, in the municipality of Janaúba at 15°48′10″ S 43°18′32″ O during the spring (as these fibers only exist during this season). It is important to note that the fibers of *Ceiba speciosa* are hollow and cylindrical with a large lumen. About 80% of these fibers are porous, distinguishing them from other natural fibers and contributing to their lightweight nature [18,19]. Furthermore, these fibers are rich in cellulose, making them an excellent raw material for producing carbon-based nanomaterials.

### 2.2. Pretreatment of Ceiba speciosa Fiber

**Figure 1:**
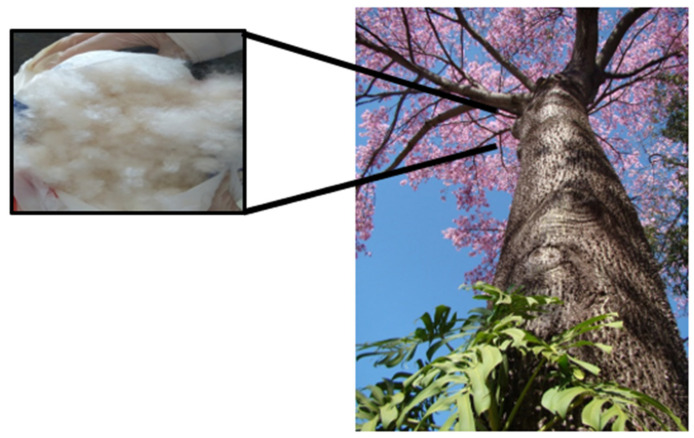
*Ceiba speciosa* and its fibers.

The fibers of *Ceiba speciosa* (Figure 1) were washed several times with DI water. After, it was dried in the oven at 60 °C for 2 h. Then, 1 g of fibers were added to 100 mL of 2% NaOH, which was maintained under moderate stirring for 3 h. Then, the fibers were washed several times with DI water until the pH reached 7.0.

### 2.3. Determination of Ceiba speciosa Fiber Chemical Composition

The chemical composition analysis of *Ceiba speciosa* fiber was conducted according to the Technical Association of the Pulp and Paper Industry (TAPPI) standards [18,19]. Briefly, 1 g of *Ceiba speciosa* fiber was added to a 72% H_2_SO_4_ solution (15 mL) and stirred for 2 h at room temperature. Following this, 560 mL of deionized water was added to the mixture, which was then stirred for an additional 4 h. The solution was subsequently centrifuged to isolate the insoluble lignin, which was dried in an oven and weighed. The lignin content was calculated using Equation (1).

(1) $$Lignin\%=\frac{M_{1}}{M}\times 100$$

where $M_{1}$ was the obtained lignin mass and M was the initial sample mass.

The amount of holocellulose was obtained by TAPPI T257 om-09, using a sodium chlorite treatment according to the literature [21]. Then, similar to the previous procedure, 1 g of *Ceiba speciosa* fiber was put into 30 mL of 10% NaCl solution, added to 0.25 mL of acid acetic, and kept at 75 °C for 1 h. After that, the solution was cooled, filtered, and washed using deionized water. The residue was dried and weighed. The obtained holocellulose amount was determined by Equation (2).

(2) $$Holocellulose\%=\frac{M_{2}}{M}\times 100$$

where $M_{2}$ was the obtained residue mass and *M* was the initial sample mass.

The amount of cellulose was determined using the residue from the first procedure, which was designed to measure the lignin content, as previously described [22]. Subsequently, this residue was mixed with a 17.5% sodium hydroxide solution in deionized water and allowed to sit for 5 h. After that, the solution was quenched using ice. The obtained white material (cellulose) was washed using deionized water until the pH became neutral. The cellulose amount was determined using Equation (3).

(3) $$Cellulose\%=\frac{M_{3}}{M}\times 100$$

where $M_{3}$ was the obtained white powder mass and *M* was the initial sample mass.

### 2.4. Cdots Synthesis

**Figure 2:**
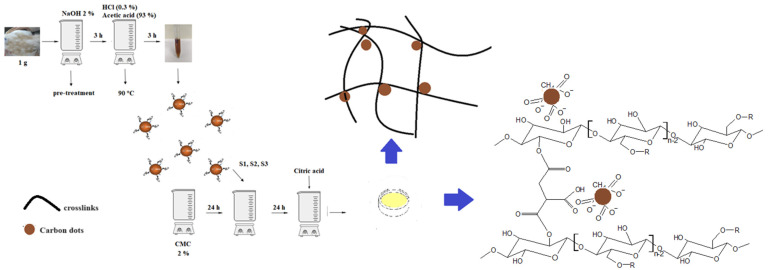
Schematic representation of the Cdots synthesis and hydrogel formation. Hydrogel with 300 µL of Cdots (S1), hydrogel with 500 µL of Cdots (S2), hydrogel with 1000 µL of Cdots (S3), and carboxymethyl cellulose (CMC).

Although most Cdots production methods rely on pyrolysis or microwave irradiation in the pursuit of greener solutions [23], these procedures are energy-intensive. This is a significant concern given current global worries about energy consumption, which the UN has frequently discussed [17]. To address these concerns, an innovative chemical method using a low-cost acid (acetic acid) and minimal energy and time was developed to reduce the production costs of Cdots. This method took advantage of the high cellulose, hemicellulose, and holocellulose content in *Ceiba speciosa* fibers, as detailed previously in Section 2.3. Unlike the common methods reported in the literature, which primarily use pyrolysis and microwaves, this new procedure employs an acid medium—a rarely used approach that is less harmful to living beings and the environment. During initial tests, only acetic acid and a small amount of hydrochloric acid at 90 °C were effective in promoting Cdot formation. Following the pretreatment steps outlined in Section 2.3, the fibers were mixed with 100 mL of 93% acetic acid and 0.3% hydrochloric acid, then maintained under moderate stirring for 3 h at 90 °C (Figure 2). In sequence, the brown solution was centrifuged 4 times at 15,000 rpm for 15 min, and the supernatant was removed, yielding a light-yellow solution. Subsequent dialysis was conducted in darkness with deionized water for 48 h using a 12–14 kDa membrane (SERVAPOR, Berlin, Germany) at room temperature to ensure the complete removal of unreacted species and water-soluble contaminants. After dialysis, the solution was transferred to plastic tubes, wrapped in aluminum foil to protect from light, and stored in a refrigerator at 4 ± 2 °C for future use.

### 2.5. Carboxymethyl Cellulose Hydrogel with Incorporated Cdots

Sodium salt of carboxymethyl cellulose (CMC) and citric acid were used as the hydrogel matrix, with Cdots added in varying proportions (Table 1). Briefly, a 2% CMC solution was prepared by dissolving CMC in deionized water under moderate stirring for 24 h. Next, the specified amount of Cdots (see Table 1) was added to the CMC solution and stirred for an additional 24 h. Citric acid (10% *w*/*w* of polymer) was then introduced and stirred for 20 min. The resulting solution was poured into a polystyrene petri dish and placed in an oven at 40 °C for 24 h to remove water, followed by heating at 80 °C to promote crosslinking of citric acid to the CMC chains, forming the hydrogel.

The values in Table 1 were determined following Transmission Electron Microscopy (TEM) analysis, which provided the Cdots concentration. This concentration was based on preparing grids and the observed Cdots count, calculated from a statistical analysis of 10 grids during TEM, yielding an average of approximately 100 Cdots in 20 µL of solution.

### 2.6. Cdots and Carboxymethyl Cellulose Hydrogel Characterization

Chemical characterization of Cdots was conducted using Fourier transform Infrared Spectroscopy (FTIR) on a Nicolet 6700 device (Thermo Fisher, Waltham, MA, USA) with attenuated total reflectance (ATR) in the wavelength range from 675 to 4000 cm^−^¹. Additionally, ultraviolet-visible (UV-Vis) spectroscopy was performed using a BEL UV-MX spectrometer (BEL, Monza, Italy) across the range from 190 to 800 nm. X-ray photoelectron spectroscopy (XPS) analysis was carried out with an Al Kα X-ray source (1486.6 eV, CTX400, PSP Vacuum Technology, Macclesfield, UK), with spectra corrected for the C1s peak (284.8 eV). The Zeta Potential was measured using a Malvern Zetasizer NanoZS (Malvern, UK) to determine the surface charge. Photoluminescence (PL) characterization of Cdots was also performed using a Horiba fluorospectrometer (Horiba, blue LED, λₑₓ = 350 ± 10 nm) at room temperature, with fluorescence intensities reported in relative fluorescence units (RFU). In addition, the Cdots were characterized by Transmission Electron Microscopy (TEM) coupled with Energy-Dispersive X-ray Spectroscopy (EDX) analyses performed on a FEI Titan G2 80-300FEG S/TEM with a Schottky-type electron gun operated at 300 kV and a Bruker XFlash 6T-30 detector (resolution 129 eV). All analyses were performed using a minimum of four replicates (*n* ≥ 4). For the characterization of Cdots, 1 mL of the Cdot solution was placed in a rotary evaporator for 24 h to allow all the solvents to evaporate, leaving only the Cdots behind. The TEM analysis was conducted as described in the previous section (Section 2.5). For the UV-Vis analysis, deionized water was used to analyze the Cdots, similar to the procedure followed for PL analysis.

In the case of hydrogels, the chemical characterization was performed by FTIR on a Nicolet 6700 device (Thermo Fisher, Waltham, MA, USA), with ATR in the wavelength range from 675 to 4000 cm^−1^. Scanning Electron Microscopy (SEM) was used for hydrogels’ characterization. Additionally, imaging was conducted using a Zeiss Ultra Plus system with accelerating voltages of 2–6 kV, a working distance of 4–5 mm, and an in-lens detector. EDX spectra were collected at 15 kV with an Oxford Inca EDX detector. X-ray diffraction (XRD) analysis was performed using a PANalytical Empyrean diffractometer (UK) with Cu-K*α* radiation (*λ* = 1.5418 Å). Measurements were taken over a 2*θ* range from 15.0021° to 69.9941° in 0.04° steps at a scan rate of 2°/min.

Swelling (SD) and gel fraction (GF) analyses were performed to assess the hydrogel’s adsorption capacity in physiological tissue conditions and its kinetic dissolution, using Equations (4) and (5) below. Briefly, the hydrogels were first weighed in their dry state (*W*0) before immersion in PBS solution (pH 7.0). After a designated period in PBS, the swollen hydrogels (*Wf*) were gently blotted with cellulose filter paper to remove excess solution from the surface and reweighed. The samples were then dried at 40 ± 2 °C for 24 h (until weight stabilization), and their final mass was recorded. To reach equilibrium for both procedures, the kinetic intervals were 1, 2, 3, 4, 6, 8, 24, and 48 h. Altogether, 21 samples were used for each system (*n* = 21, seven samples of three different syntheses). All results were statistically equivalent (ANOVA, one way included Tukey’s test, *p* < 0.05, software Origin v.8.1, OriginLab Corporation, Northampton, MA, USA). A similar procedure was also performed using a physiologic simulated body fluid (SBF) medium prepared in the laboratory.

(4) $$SD=[(W_{f}-W_{0})/W_{0}]\times 100\%$$

where *W_f_* is the weight of the swollen polymer after drying, and *W*_0_ is the initial weight of the polymer.

(5) $$GF=[1-[(W_{0}-W_{f})/W_{0}\;]]\times 100\%$$

where *W_f_* is the weight of the swollen polymer after drying, and *W*_0_ is the initial weight of the polymer.

### 2.7. Biological Analyses of Carboxymethylcellulose Hydrogel with Cdots Incorporated

#### 2.7.1. Cell Culture

Human cervical carcinoma cells (HeLa, American Type Culture Collection—ATCC@CCL-2) were provided by Prof. Maria de Fátima Leite from the Department of Physiology and Biophysics at the Federal University of Minas Gerais. These cells were chosen because they are usual model cells used to explore other female diseases, such as human papillomavirus (HPV) [24]. The cells were cultured in ‘Dulbecco’s modified eagle medium’ (DMEM), with 10% (*v*/*v*) fetal bovine serum (FBS) and streptomycin sulfate (10 mg·mL^−1^), sodium penicillin G (10 units·mL^−1^), and amphotericin-b (0.025 mg·mL^−1^), all supplied by Gibco BRL (Grand Island, NY, USA), in an oven with 5% CO_2_ and a temperature of 37 °C.

#### 2.7.2. Cell Viability Assay with Resazurin

HeLa cells were trypsinized and plated (3 × 10^4^ of cells /well) in 24-well plates. As a reference control, cells plus culture medium (DMEM + 10% FBS) were used, and as a positive control, Triton X-100 (2% *v*/*v* in phosphate buffered saline; PBS, Gibco BRL, Grand Island, NY, USA). Hydrogel samples were used per well, using 500 μL of culture medium to complete the well volume. After 24 h of treatment, the present medium was aspirated and replaced by a culture medium solution (DMEM + 10% SFB) with 10% of the resazurin solution in PBS, in the proportion of 5%, and incubated for 3 h in an oven with 5% CO_2_, at 37 °C. Then, 100 μL of this solution was transferred to a flat 96-well plate and submitted to spectrophotometric analysis at 530 nm and 590 nm wavelengths using Varioskan equipment (Thermo Fisher). The cell viability was measured using Equation (6).

(6) Cell viability(%)=(Abs of sample and cells/Abs of control cells)×100

#### 2.7.3. Cellular Uptake of Hydrogels by Laser Scanning Confocal Microscopy

HeLa cells were plated on coverslips (3 × 10^5^ of cells /well) in a 6-well plate. After 48 h, the cells were treated with 400 μL of medium (DMEM + 10% SFB) and 1 cm^2^ of each hydrogel type (Table 1), with treatment times of 30 and 60 min. Cells in culture medium (DMEM + 10% SFB) without treatment were used as controls. After treatment, the cells were fixed with 4% paraformaldehyde (Electron Microscopy Sciences, Hatfield, PA, USA). The slides were mounted with Hydromount (National Diagnostics, Atlanta, GA, USA) and analyzed under a confocal microscope. Images were captured on an Eclipse Ti confocal microscope (Nikon Instruments, Melville, NY, USA) using an oil immersion objective (63 × Plan-Apo/1.4 NA) with green emission (λexc = 488 nm and λem = 506–550 nm), collected with a FITC filter. For the Cdots and hydrogels analysis using this technique, deionized water was used, and hydrogels without solvent were added to the medium described above.

### 2.8. Statistical Analysis

The statistical analysis was performed using ANOVA (one way included Tukey’s test, *p* < 0.05, software Origin v.8.1, OriginLab Corporation, Northampton, MA, USA) unless specifically noted.

## 3. Results and Discussion

### 3.1. Chemical Composition of Ceiba speciosa Fiber

To determine the chemical composition of *Ceiba speciosa* fibers, TAPPI norm T222 om-88 was performed [21,22]. Our results demonstrated that the composition of holocellulose (91 ± 4)%, cellulose (61 ± 11)%, hemicellulose (9 ± 1)%, and lignin (5 ± 1)% were higher than the other holocellulose and cellulose compared with values reported in the literature from other natural fibers [25,26]. These results are justified by the origin of fiber, the climate of the semiarid region, and the soil source. Additionally, the composition of these fibers suggests their high potential for producing carbon-based nanomaterials, such as Cdots. Mwaikambo and Ansell [27] reported that jute fiber consists of 67% cellulose, 16% hemicellulose, and 9% lignin, while cotton fiber contains 89% cellulose, 4% hemicellulose, and 0.75% lignin, and bagasse fiber comprises 37% cellulose, 21% hemicellulose, and 22% lignin. Waikambo [28] noted different compositions in nettle fiber, with no lignin present and proportions of 85% cellulose and 10% hemicellulose. These variations underscore that the composition of natural fibers can vary significantly depending on the region, type of fiber/source, soil, and climate conditions.

### 3.2. Spectroscopy Analysis

**Figure 3:**
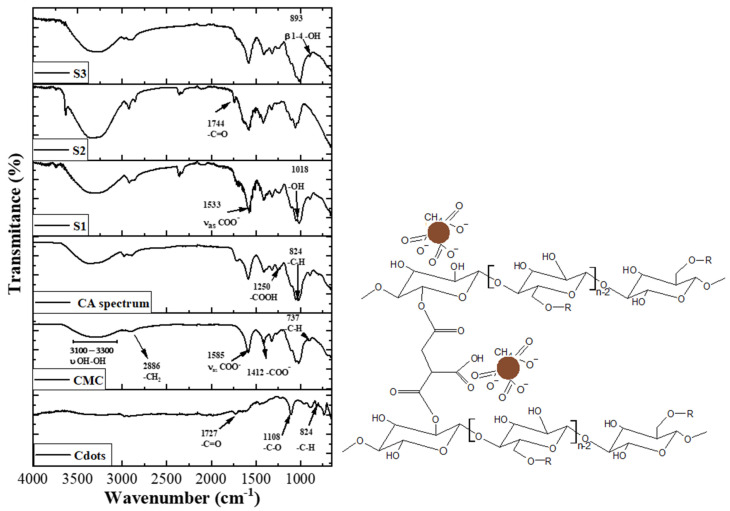
ATR-FTIR spectra of Cdots, CMC, and hydrogels (CA, S1, S2, S3) (inset, CMC linked with CA; the brown circles are the Cdots).

In the ATR-FTIR spectra of Cdots, the signal was perceived at 1727 cm^−1^, 1108 cm^−1^, 824 cm^−1^, and 737 cm^−1^, related to -C=O, -C-O stretching, and -C-H vibrations, respectively, associated with carboxyl groups and others stabilizing the Cdots (Figure 3) [29,30,31]. These results indicate that cellulose, hemicellulose, and holocellulose are found in the fiber, suggesting that the chemical groups in cellulose, hemicellulose, and holocellulose stabilize the Cdots. In addition, it was perceived that bands at 3100–3300 cm^−1^ were associated with hydroxyl groups in CMC. In hydrogels (including the hydrogel without Cdots), the signal of this band was increased when compared with pure CMC due to the crosslinking between the citric acid and CMC, as schematically represented in Figure 3 (inset) [29,30,31]. Additionally, this band was higher in sample S2, compared with samples S1 and S3. We hypothesize that in the case of sample S2, most of the Cdots linked with citric acid before this acid crosslinked with CMC, making a bridge, which in this sample improved the intensity of the band associated with OH groups. Indeed, the Cdots were added to the CMC solution before the citric acid and in higher concentrations for sample S1. In the case of sample S3, the highest concentration of Cdots, this was not observed, mostly due to the highest concentration of Cdots with a negative charge on the surface, promoting repulsion among them and not affecting the crosslinking between CMC and citric acid. Additionally, bands at 2984, 2930, and 2916 cm^−1^ corresponding to CH_2_ stretching are observed, with a blue shift in the hydrogels compared to CMC_CA (carboxymethyl cellulose hydrogel with citric acid). This shift suggests a stronger interaction within the CMC_CA hydrogel matrix and a red shift that intensifies with increasing Cdots concentration, enhancing the nanocomposite’s luminescent properties [16,29,30]. Furthermore, at 1570, 1570, and 1578 cm^−1^, bands associated with the carboxylate group are perceived to have a slight displacement to the blue region compared to hydrogels without Cdots (CMC_CA) and pure CMC. In addition, this band increases the signal when the Cdots concentration increases, mainly due to the presence of this group at the Cdots surface. Furthermore, bands associated with polysaccharide rings O-H and C-H bending vibrations close to 1200 cm^−1^ and C-O vibrations from primary and secondary alcohols were perceived in all samples [16,29,30], and bands related to the carbonyl group (C=O) are visualized in hydrogels but not in pure CMC.

**Figure 4:**
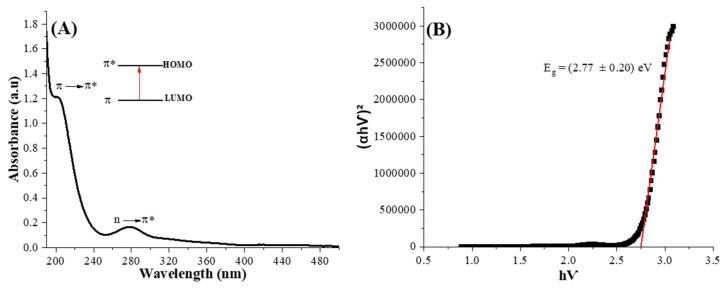
UV visible spectra of Cdots (**A**) and Tauc relation of Cdots (**B**).

The ultraviolet-visible spectra are shown in Figure 4. At 204 nm and 278 nm, it is perceived that two bands are associated with π-π* (linked core sp^3^ hybridization), n-σ*, and n-π* (surface functional groups), respectively, chiefly linked to C = C and hydroxyl groups [32,33,34]. In addition, the band gap (Eg) was calculated using the Tauc method, yielding a value of 2.77 ± 0.20 eV, which falls within the band gap range typical of semiconductor materials and corresponds to the blue light energy spectrum [35]. The value was similar to other studies found in the literature for this range of Cdots (1.5–3.5 eV) [36,37].

**Figure 5:**
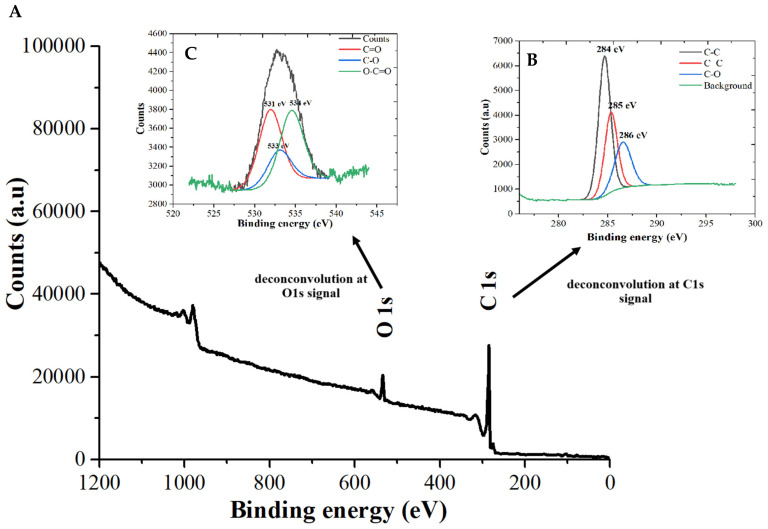
(**A**) X-ray Photoelectron spectroscopy (XPS) spectrum of Cdots, (**B**) C1s deconvolution, and (**C**) O1s deconvolution.

In the X-ray Photoelectron Spectroscopy (XPS) analysis (Figure 5), the proposed technique was to determine the elements, their links with others in each orbital, and their oxidate state. However, it is important to perform the deconvolution of each signal perceived for each element and orbital to reach these results. Then, in this case, the main elements are carbon and oxygen. Thus, deconvolution using Gaussian curves with the software OriginPro 2023 was performed on the C1s and O1s spectra to identify the chemical groups on the Cdots’ surface. The C1s deconvolution revealed three main peaks at binding energies of 284 eV, 285 eV, and 286 eV, corresponding to C–C bonds with sp^3^ hybridization and C–O bonds with sp^2^ hybridization, respectively. The results indicated a high presence of hydrophilic groups on the Cdots’ surface, suggesting excellent water solubility. In addition, the results found in the XPS analysis confirmed the UV-visible results that are π-π* graphitized core sp^3^ hybridization and n-π* in the surface [32,33,34]. In addition, it was observed that the three main peaks of O atoms (O1s) at 531, 533, and 534 eV were very close to the results found in the literature [38]. These peaks imply that the oxygen appeared in O-H, C=O, and C-O, respectively [39,40]. The intensity of signals in the O1s analysis indicates that the OH group has more exposure to the edge’s sites, implying that the structure is defect-free [39,40].

### 3.3. Photoluminescence Analysis

**Figure 6:**
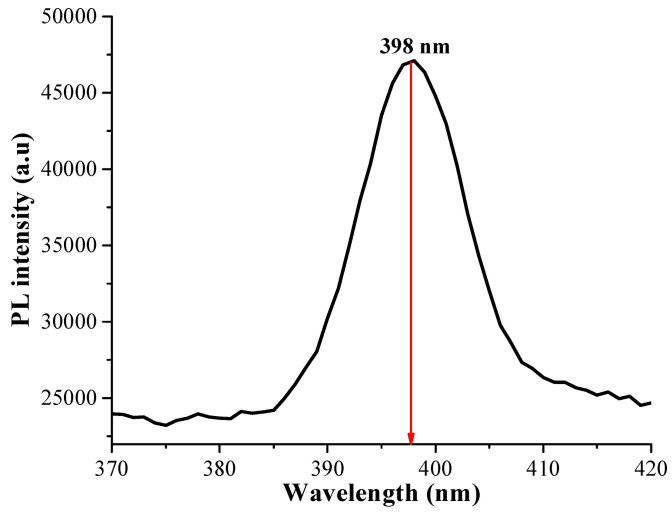
Photoluminescence (PL) spectra of Cdots. The peak photoluminescence was observed at 398 nm.

The PL spectroscopy analysis is shown in Figure 6. The obtained spectra allowed us to determine the quantum yield (*QY*) using quinine rhodamine as a standard. The Cdots solutions were used *n* = 1.33. The *QY* of the Cdots was obtained from Equation (7) based on literature [41]. The *QY* of Cdots was 11%.

(7) $${QY}_{Cdots}={QY}_{SD}\left[\frac{{\left(\frac{dI}{dA}\right)}_{Cdots}}{{\left(\frac{dI}{dA}\right)}_{SD}}\right]\times (\frac{n_{Cdots}^{2}}{n_{SD}^{2}})$$

where ${QY}_{SD}$ is the quantum yield of Rhodamine, *I* is the area of PL spectra, and *A* is the maximum absorbance in the PL spectra.

The idea of employing carbon quantum nanomaterials (Cdots) in biomedical applications is relatively recent. Their unique and promising optical properties have garnered significant scientific interest, highlighting their potential for use in drug delivery systems coupled with the diagnosis of various diseases, including uterine cancer. This potential represents an innovative advance in the field of materials science, particularly in the area of theranostic analysis, which is a novel concept [42]. The Cdots are known for their intrinsic luminescence, which gives them a high potential for various applications in different sectors, such as bioimaging [43], photocatalytic oxidation [44], metal detection [45], analytical chemistry [46], renewable energy [47], and others. PL spectra identified a central band at 398 nm (close to blue light). Nontraditional luminogens have already been described in the literature [48,49]. These intriguing materials, primarily polymers, exhibit luminescence properties despite not being small nanoparticles. This is due to their production process, chemical and polyelectrolyte groups, and the ability to conjugate with other nanomaterials [48,49]. In this context, our research involved incorporating Cdots at various concentrations to assess their potential in bioimaging and therapeutic applications, particularly in treatments related to women’s health. Furthermore, the addition of CMC combined with citric acid may enhance the luminescence properties, thereby broadening these applications, given that CMC is a recognized polyelectrolyte [16,50]. The PL of hydrogels cannot be performed, as the crosslinking did not allow the hydrogel to be dissolved in a solvent.

### 3.4. Morphological Analyses

**Figure 7:**
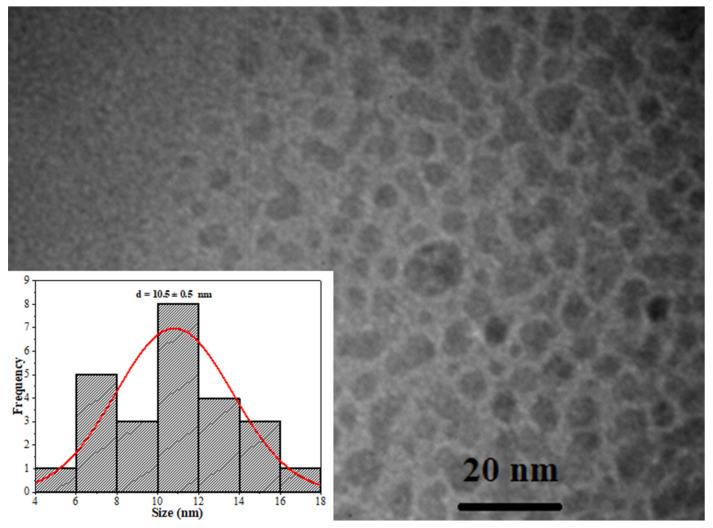
TEM microphotograph of Cdots and its diameter distribution, obtained using ImageJ software 1.54 from TEM images (red line is the gaussian curve of histogram).

TEM and SEM analyses were conducted to determine the size of the Cdots and their distribution within the hydrogels and to carry out other essential morphological assessments. Figure 7 displays the TEM images of Cdots. The morphological analysis showed that the average size of the Cdots is 10.5 ± 0.5 nm, which aligns closely with sizes reported in previous studies [41,48]. This is a significant finding, considering the aim of this research to apply Cdots in cellular bioimaging for potential diagnostics, as smaller sizes may enhance cellular internalization. Furthermore, the charge on the Cdots’ surface, which can influence endocytosis in cells [16], was evaluated using Zeta Potential measurement. The measured zeta potential was −19.5 ± 0.2 mV, indicating a negative surface charge with a polydispersity index (PDI) of 0.448. As highlighted in the spectroscopic analyses above, this negative charge is primarily due to the presence of carboxylate (COO^−^) groups on the Cdots surface.

**Figure 8:**
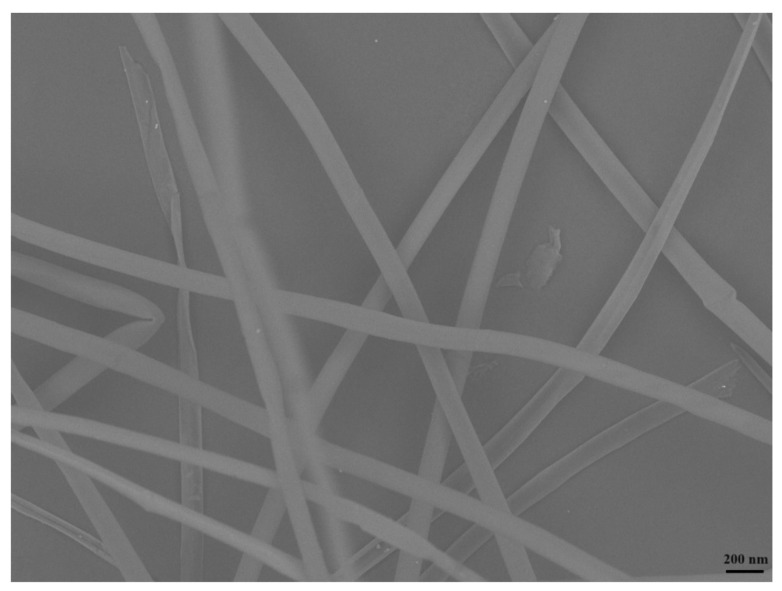
SEM images of *Ceiba speciosa* fibers with a scale bar of 200 nm.

In numerous biomaterial applications, promoting strong cell adhesion is crucial. For diagnostic and therapeutic applications, proper membrane adhesion is a key factor for the effective internalization of the luminescent biomaterial [16,47,51]. In addition, the homogeneous distribution of the nanoparticles in the hydrogel can increase these characteristics [16]. Considering these aspects, assessing new biomaterials requires a detailed evaluation of morphology and surface characteristics. Therefore, SEM analyses were performed on the hydrogels (Figure S1). In a close look into the images, it is possible to observe the Cdots embedded by the hydrogel. Such embedding makes the observation of Cdots by SEM not so evident when loaded into the hydrogels. Additionally, SEM images of *Ceiba speciosa* fiber before and after being treated were collected. The SEM images showed that the Cdots were distributed homogeneously in the material, as no clusters were perceived in the hydrogels. In addition, the hydrogels seemed regular without any roughness. In the case of the fibers, the images before showed a fiber with rough edges and a diameter close to 200 nm. After being treated, the fibers appear to be smoother (Figure 8).

Table 2 presents a comparison of Cdots derived from various sources. Most Cdots described in the literature are synthesized using chemical molecules like citric acid or carbohydrate sources, primarily sourced from the chemical industry. Few studies focus on natural sources, with the majority using citrus fruits as a citric acid source for Cdot synthesis. Consistent with the findings in this research, the size of Cdots reported in the literature typically ranges from 2 to 10 nm. Additionally, several studies [17,52,53,54,55] indicate that the morphology of Cdots can alter their optical properties. The applications of these Cdots are diverse, extending from optical to biomedical uses, including cancer diagnosis, as detailed in Table 2. Additionally, the studies listed in Table 2, though employing green synthesis methods, did not use natural fibers. Furthermore, their synthesis processes were complex, involving multiple steps that complicate their reproducibility. Despite these differences, the chemical composition of the Cdots’ surfaces in these studies is very similar to ours, predominantly featuring C=O, C-H, and OH groups.

### 3.5. XRD Analysis

**Figure 9:**
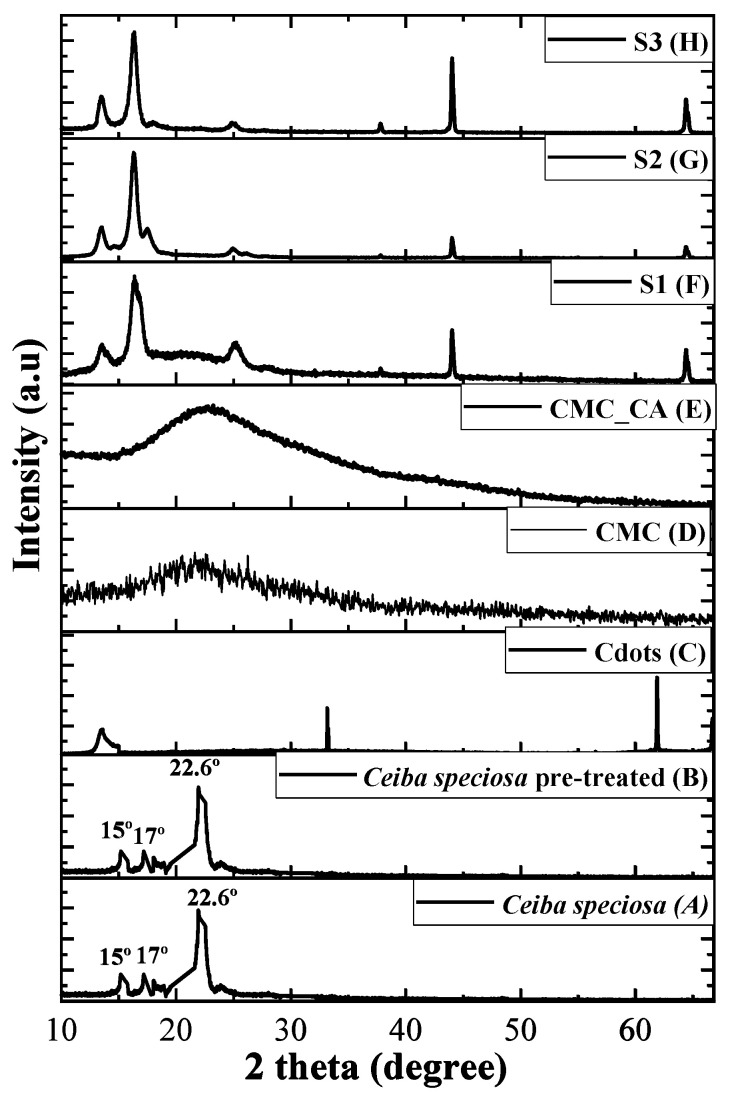
XRPD patterns of (**A**) *Ceiba speciosa* before and (**B**) after being treated; (**C**) Cdots; (**D**) pure CMC and hydrogels (**E**) CMC_CA; (**F**) S1; (**G**) S2; (**H**) S3.

The XRD analyses of hydrogels, Cdots, *Ceiba speciosa* fibers before and after treatment, and pure CMC are shown in Figure 9. The XRD diffractogram presented four main peaks at 14°, 16°, 44° and 65° in the hydrogels with Cdots, three in the Cdots at 14°, 33° and 62°, and one in pure CMC at 22° and hydrogel CMC_CA at 23°. According to the literature, these peaks in the Cdots depend on their crystallinity, which depends on the synthetic methodology and source used (e.g., natural fiber) [16,56]. The diffractogram of pure CMC and CMC_CA showed an amorphous structure, justifying a slight change perceived between them in the main peak. This behavior is caused by a cross-link between CMC and citric acid, which may cause an amorphization or shift in the main peak of CMC [16,56]. In *Ceiba speciosa* fibers, a peak at 22.6° and three broad peaks at 15°, 17°, and 34.3° related to cellulose I; in addition, the prominent peak at 22.6° is related to the pattern (0 0 2) [57]. In the case of Cdots, the synthetic methodology used and the source selected provided Cdots that are very crystalline when compared with examples previously reported in the literature [17,33].

In addition, the crystallinity index was determined using the “peak deconvolution” [48,58,59] using the Origin2022b software (Figure S2, Supplementary Materials). The crystallinity index was performed using this amorphous method, and the results were 79%, indicating that these Cdots possess high crystallinity, probably because of the high cellulose and holocellulose content in the fibers, as described by TAPPI norm T222 om-88. Consequently, a significant difference in crystallinity is observed in the hydrogels containing Cdots. New peaks associated with rhombohedral carbon appeared at 14° and 44° (ICDD 00-048-1449). In addition, a peak related to the graphene structure appeared at 65° [60]. Moreover, the main peak at 22° in the CMC suffers a shift to 16°. This modification may happen because of the interaction between chemical groups at CMC and the chemical groups on the Cdot surface promoted by the heat treatment used to produce the hydrogels [61,62]. In the literature, it is described that some carbon structures appear with heat treatment [17,56]. This behavior can be explained by heat treatment, which forms new bonds between the chemical groups of CMC and Cdots (Figure 9 inset, Equation (8)). In the case of the fibers, it was observed new peaks after treatment with NaOH related to the presence of base residues which are not completely removed in the washing step. Therefore, the peaks presented in the *Ceiba speciosa* fibers before and after being treated are similar to cellulose peaks described in the literature [59,62]. Additionally, the Scherrer equation (Equation (9)) [62,63] was applied to determine the crystallite size of the Cdots, calculated to be 3.2 nm.

(8) $${CMC}_{COOH}^{:OH}\overset{pka\geq 4.5}{\to }{CMC}_{{COO}^{-}}^{:OH}+{Cdots}_{{COO}^{-}}^{{CH}_{4}}\to [{CMC}_{{COO}^{-}}^{OH}/{Cdots}_{COOH}^{OH}]$$

(9)  D=kλ/βcosθ

where *D* is the diameter in nanometers (nm) of the nanoparticles; *k* is the Scherrer constant (0.89); *λ* = 0.15418 nm; *β* is the average peak width; and *θ* is the Bragg diffraction angle corresponding to the Cdots reflection.

### 3.6. Swelling Degree and Gel Fraction Analysis

**Figure 10:**
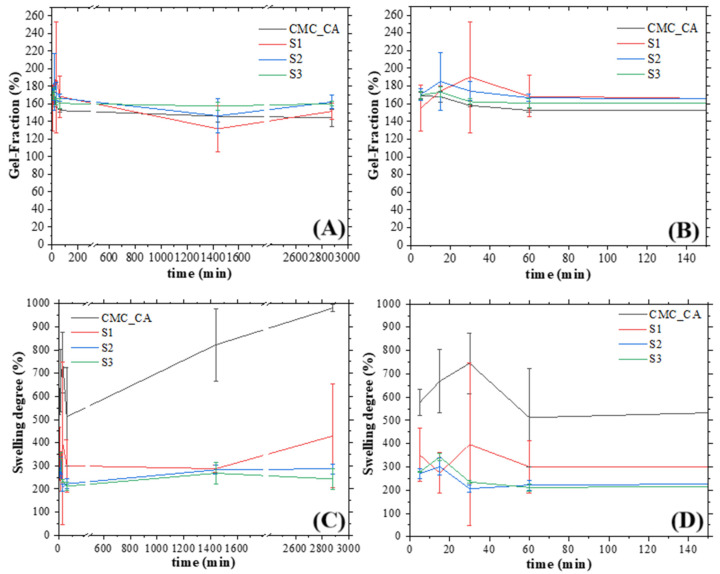
(**A**) Gel fraction of hydrogels; (**B**) Enlarged view of the gel fraction graph for the initial minutes; (**C**) Swelling degree of hydrogels; (**D**) Enlarged view of the swelling degree graph for the initial minutes.

One important parameter of biomaterials is the adsorption of physiologic fluid and the degradation or dissolution in the physiological medium. Considering that, the swelling behavior and gel fraction (dissolution into medium) were performed. The results are presented in Figure 10A (gel fraction, GF) and B (swelling degree, SD). In the case of GF, the behavior of all hydrogels was similar, and the chemical stability was very good. Nevertheless, in the case of SD, the hydrogel without Cdots showed more adsorption than hydrogels with Cdots, but non-statistically significant differences were noted in hydrogels S1, S2, and S3. These results showed that the presence of Cdots in the structure of hydrogel decreased the adsorption behavior of CMC-CA, mainly when the Cdots concentration increased, which was expected, as some functional groups in the CMC were crosslinked with the Cdots, and therefore less available to connect with adsorption sites. In addition, as previously described in the XRD analysis, the presence of Cdots in the hydrogel networks made their structures more crystalline. With a more rigid and organized tridimensional structure, we improve chemical stability and decrease fluid absorption by the biomaterial. Moreover, the results herein reported were aligned with those already reported in the literature [64,65]. Similar results were found for swelling and gel fraction performed with SBF medium (Figure S3).

### 3.7. Biological Analysis

Ultrasmall carbon quantum dots (Cdots) have a range of applications, including cell labeling and cancer cell diagnosis. These nanomaterials show great potential as theranostic materials, especially when combined with polyelectrolyte polymers such as polysaccharides [42]. They have also been explored as effective nanoprobes for bioimaging and drug delivery, owing to their optical and electrical properties and the ability to conjugate their surface chemical groups with drugs for various diseases [16]. Modern techniques in cancer cell imaging, which are crucial for diagnostics, surgical guidance, and interpreting treatment outcomes, often distinguish between normal and neoplastic tissues. Research in this field aims to develop tools that combine diagnostic and treatment capabilities, termed theranostic materials [42]. Therefore, Cdots could represent a significant advancement in cancer cell fluorescent imaging [16,42]. Cdots are nanomaterials with significant photoluminescence and a size of less than 10 nm, making them extremely promising for modern medicine. CDots have several potential applications, such as bioimaging, sensors, and drug delivery [64,66]. Therefore, it becomes extremely important to analyze the viability and applicability of this nanomaterial, considering that Cdots have advantages such as their high solubility in water and photoluminescence, although they can be suspended, low toxicity, high photostability, and functionalization possibilities [64,66].

Additionally, in the case of hydrogels, several examples have been reported considering the performance of these materials due to their promising features, such as mechanical performance, biodegradability, chemical stability, and tissue compatibility [67]. Considering that the development of these polymeric matrices to improve drug delivery based on the fine tuning of their physicochemical parameters, allowing better dosage control and pharmacokinetic behavior is a fast-growing field in biomedical sciences and engineering [67,68]. Moreover, the hydrogels that incorporate micro- and nanoparticles into their matrix can enhance other crucial characteristics, such as mechanical behavior and thermal, optical, and electrical properties, and produce nanocomposites [16]. Therefore, to assess the potential of the Cdots developed in this study for bioimaging applications, particularly in potentially diagnosing uterine cancer and potential theragnostic uses, tests were conducted to evaluate cellular viability and uptake using HeLa cells.

**Figure 11:**
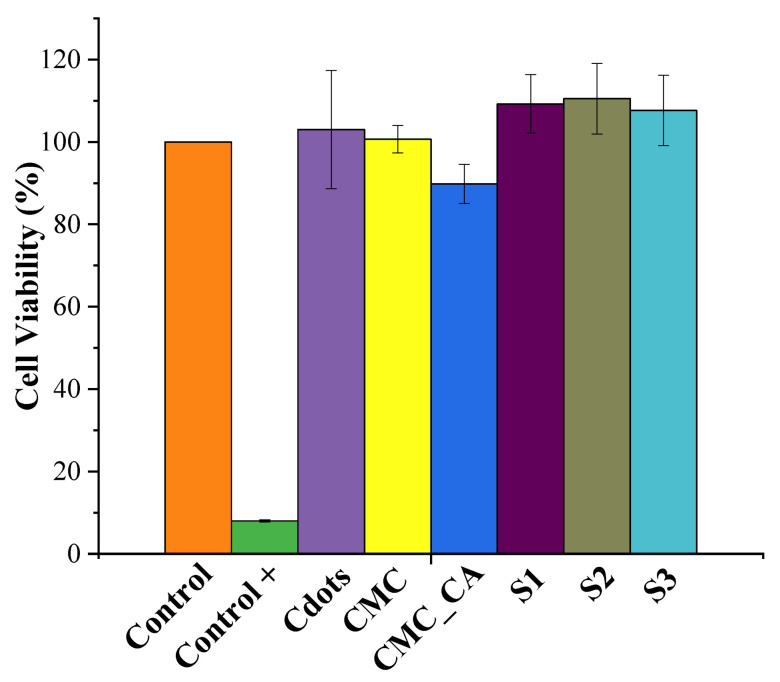
HeLa cells viability measured by resazurin assay, for Cdots, CMC, and hydrogels (CMC_CA, S1, S2, S3). Controls are cells without any other material, and control+ is triton).

Then, cell viability of Cdots, CMC, and hydrogels with and without Cdots was assessed using an assay with resazurin in HeLa cells, evaluating the cytotoxicity of the nanomaterial. Figure 11 shows that as expected, the positive control, containing Triton X-100 (a non-ionic surfactant for cell lysis), was the only one that showed a significant difference compared to the reference control (*p* < 0.01, ANOVA, Tukey’s test). Cdots samples, CMC, and hydrogels, when in contact with HeLa cells, did not show toxicity compared to the control group (*p* < 0.01, ANOVA, Tukey’s test). Furthermore, the incorporation of Cdots into the hydrogels did not generate toxicity at any concentration, indicating that these hydrogels have potential for biomedical applications without posing harm to women’s health. Nevertheless, further testing in various cell types, including healthy cells, should be conducted.

**Figure 12:**
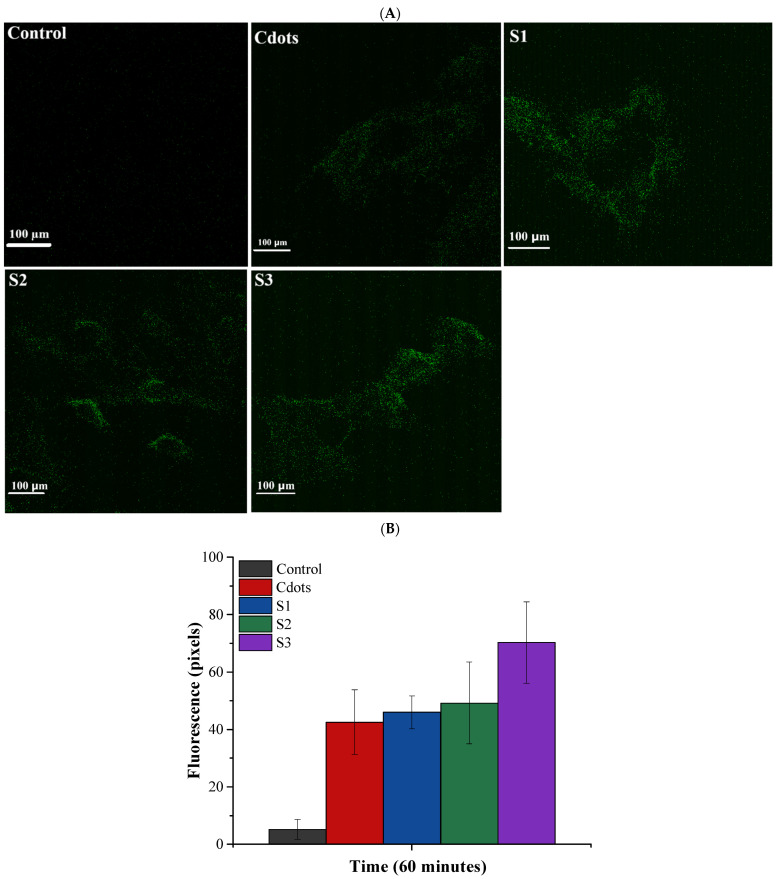
(**A**) Confocal laser scanning microscopy images showing cellular uptake of Cdots and hydrogels (S1, S2, S3); (**B**) Fluorescence results post internalization, displaying the average fluorescence intensity measured after 60 min (n = 3).

The cellular uptake of hydrogels was assessed using laser scanning confocal microscopy combined with a spectrophotometer for Cdots and hydrogels (S1, S2, and S3). Human cervical carcinoma cells (HeLa) were used to evaluate internalization, as these cells are a model for cervical cancer [22,69]. The images obtained are depicted in Figure 12, which demonstrates the time-dependent internalization of Cdots and hydrogels in HeLa cells. From the images, the mean fluorescence intensity was quantified for each time point and type of treatment. Three images of each sample were used to evaluate the average and standard deviation of internalization at each time point, shown in Figure 12B. The hydrogels had a slightly higher luminescence than pure Cdots, and a slight difference is perceived when the Cdots concentration increases in the hydrogel. These findings suggest that incorporating Cdots at various concentrations enhances the capabilities for bioimaging and therapeutic applications, aligning with this study’s aim. CMC combined with citric acid is proposed to enhance luminescence properties, thereby broadening potential applications. This is significant as CMC is a well-known polyelectrolyte recognized for its nontraditional luminogenic properties [16,49,50]. Nontraditional luminogens, which are interesting materials primarily composed of polymers, exhibit luminescence properties despite not being small nanoparticles. This luminescence is attributable to their production methods, chemical functional groups, polyelectrolyte characteristics, and the ability to conjugate with other nanomaterials [16,49]. In this study, considering the photoluminescence of CMC (Figure S4) and the wide Cdots band gap observed in the UV-Vis section based on the Tauc relation, various Cdots concentrations were incorporated into the CMC hydrogel to enhance the nanocomposite’s luminescent properties. This approach aims to increase its potential for bioimaging in theranostic applications, as higher luminescence correlates with improved fluorescence intensity, which can enhance diagnostic imaging capabilities.

**Figure 13:**
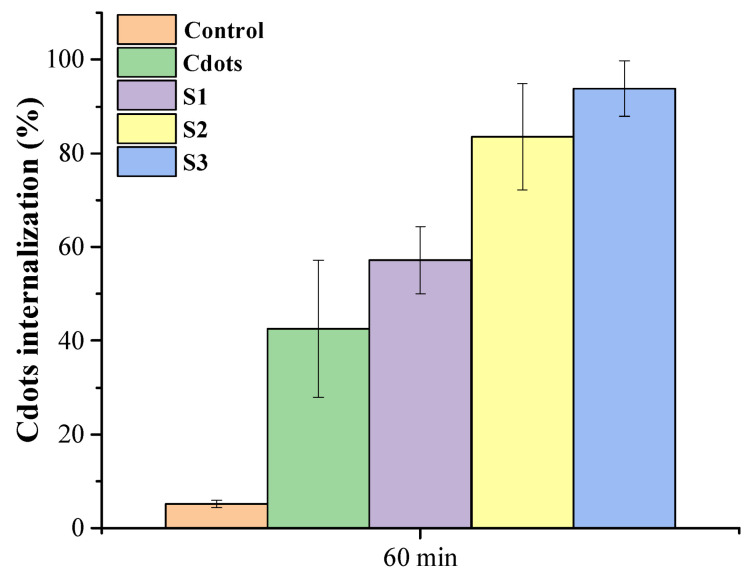
Percentage of Cdots internalization in HeLa cells after 60 min of exposure.

Additionally, after 60 min of contact between the Cdots and hydrogels, spectrophotometric analysis revealed a significant difference compared to the control (*p* < 0.01, ANOVA), highlighting the effectiveness of the nanomaterial. However, a slight statistical difference was noted among the S3 hydrogel samples (*p* < 0.01, ANOVA, Tukey’s test). These results confirm the potential of using these hydrogels as therapeutic biomaterials for women’s health. Considering the significant potential of these hydrogel nanocomposites, which are based on a polysaccharide (carboxymethyl cellulose) and loaded with Cdots, for theragnostic applications, future modifications could include the incorporation of specific drugs for uterine cancer treatment. Additionally, there is potential for chemical modifications on the Cdots’ surface to make them less harmful to the human biological environment. This is especially relevant given the enhancement of their luminescent properties. Additionally, the results of the Cdots internalization over time (60 min) are displayed in Figure 13. The results indicate a high level of internalization with an increased concentration of Cdots in the hydrogel. This demonstrates the significant potential of this luminescent nanomaterial for use in cancer diagnosis through bioimaging analysis. The cellular internalization of Cdots conjugated with carbohydrate hydrogels occurs through the linkage of chemical groups in the hydrogels with those on the cell membrane, as the hydrogels possess chemical groups similar to those found in cell membranes. Following this conjugation, the hydrogels release the Cdots into the cells, enhancing cellular luminescence, predominantly in the cytosol rather than the nuclei, although it is present in both cellular regions. This behavior is attributed to the fact that tumor cells exhibit a faster metabolism than healthy cells [16,70,71]. Furthermore, the internalization process is facilitated by endocytosis, which occurs due to interactions between the charges and chemical groups on the surface of the Cdots. This internalization is associated with toxicity; however, since Cdots are nontoxic and have chemical groups similar to those of the cell membrane, the cells do not initiate a defense response against this quantum nanomaterial. This leads to a high rate of Cdots internalization in HeLa cells. Moreover, although the QY observed in this study is relatively low compared to others in the literature [72], the rapid and extensive internalization of Cdots in HeLa cells—nearly 100% within one hour (*p* < 0.01, ANOVA, Tukey’s test)—suggests that these nanomaterials have substantial potential for use as theragnostic agents in future in vivo studies.

Furthermore, compared to results from other Cdots derived from different sources [73,74], the fluorescence outcomes—especially in the S3 samples—demonstrated superior performance. This highlights the significant potential of *Ceiba speciosa* to produce innovative, highly luminescent Cdots suitable for theranostic applications, particularly for uterine diagnostics in women’s health.

## 4. Conclusions

This work described the production of new CMC hydrogels with the incorporation of different concentrations of new Cdots obtained from a new Brazilian semiarid biomass source for potential applications in bioimaging. Our results showed that the Cdots changed the structure of hydrogels and their crystalline structure, mainly because of heat treatment and the interaction among functional groups on the CMC and Cdots surfaces. In addition, these hydrogels suffer red and blue shifts in the presence of Cdots. It was also observed that the Cdots were homogeneously distributed within the hydrogel matrix. Furthermore, the cytotoxicity evaluation performed using the resazurin assay showed good tissue biocompatibility of hydrogels in the presence or absence of Cdots. The cellular uptake experiments showed intracellular fluorescence of the Cdots and hydrogels after incubation with HeLa cells. Additionally, the concentration of Cdots in hydrogels showed a significant difference in fluorescence, even compared with pure Cdots. These results confirm the potential of focusing these hydrogels as therapeutic and/or diagnosis tools.

## Figures and Tables

**Table 1:** Hydrogel composition.

Hydrogel	Carboxymethyl Cellulose (CMC) (%)	Citric Acid (CA) (%)	Cdots (µL)
Hydrogel without Cdots (S0)	2	10	0
S1	2	10	300
S2	2	10	500
S3	2	10	1000

**Table 2:** Comparison of Cdots produced in the literature.

Source of Cdots	Size (nm)	Band Gap (eV)	Application	Reference
Citric acid monohydrate	3.4–9.5	2.4–2.5	Optical applications	[39]
Glucose	1.5–5.0	-	Pancreatic cancer	[15]
Sodium tetraborate decahydrate	3.0–4.0	-	Sensitive detection of borax	[50]
Citric acid	2.8	-	DNA damage detection	[51]

## Data Availability

The authors confirm that the data supporting the findings of this study are available within the article and/or, if necessary, to share.

## Supplementary Materials

The following supporting information can be downloaded at https://www.mdpi.com/article/10.3390/pharmaceutics16121500/s1: Figure S1. Deconvolution of Cdots. Figure S2. Deconvolution of the Cdots XRD peaks (A, B, and C) was performed using Lorentzian fitting with OriginPro 2023. Panel D shows the deconvolution applied across all XRD diffractograms. Figure S3. (A) Gel-Fraction of hydrogels in SBF (B) Representing a zoom in the first minutes of Gel-Fraction analysis of hydrogels; (C) Swelling degree of hydrogels in SBF; (D) Representing a zoom in the first minutes of Swelling analysis of hydrogels. Figure S4. Photoluminescence of CMC at 405 nm, demonstrating that pure CMC exhibits luminescent properties, which may enhance the luminescence of the hydrogel when combined with Cdots.
