# Supplementary material for: Sustainable Carbon Dots Loaded into Carboxymethylcellulose Based Hydrogels for Uterine Cancer Bioimaging
Source: Pharmaceutics. 2024 Nov 22;16(12):1500. doi: 10.3390/pharmaceutics16121500 (PMC11677459; doi:10.3390/pharmaceutics16121500)
Supplement: Supplementary file 1 [file pharmaceutics-16-01500-s001.zip › pharmaceutics-3274637-supplementary.pdf]

Supplementary Materials

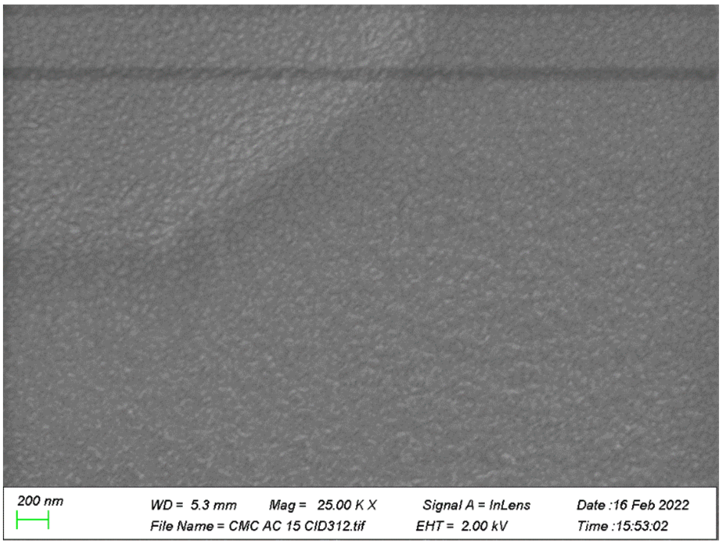

(A)

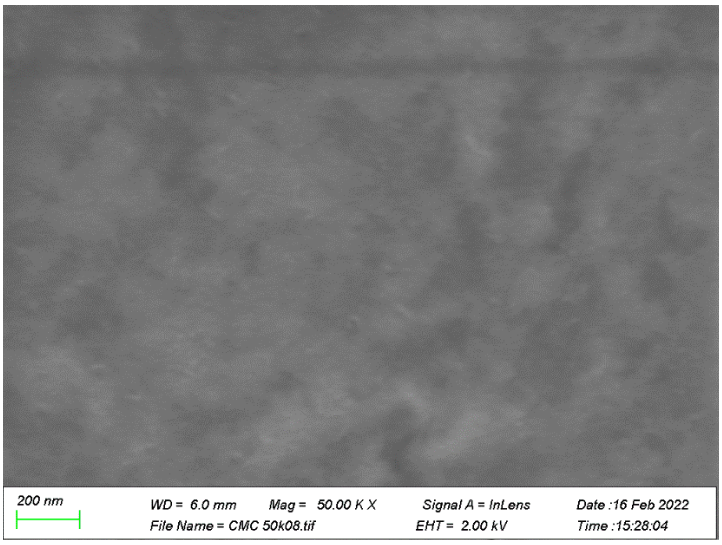

(B)

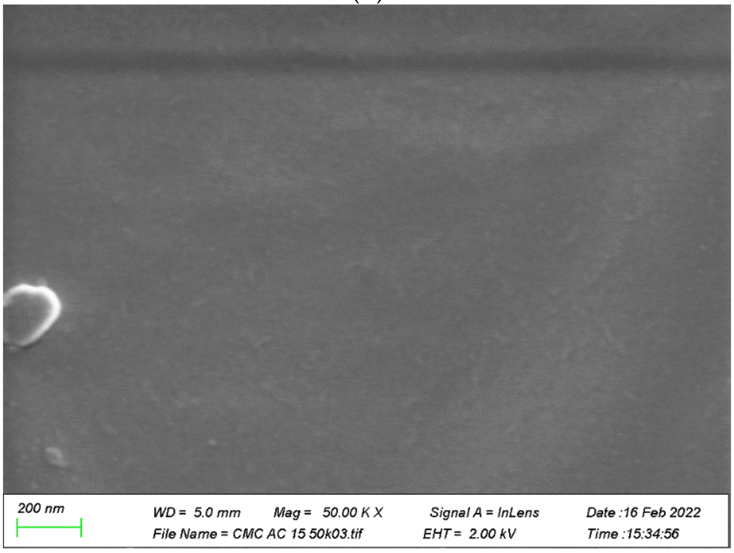

(C)

Figure S1. SEM images of hydrogels (A) S1, (B) S2, (C) S3

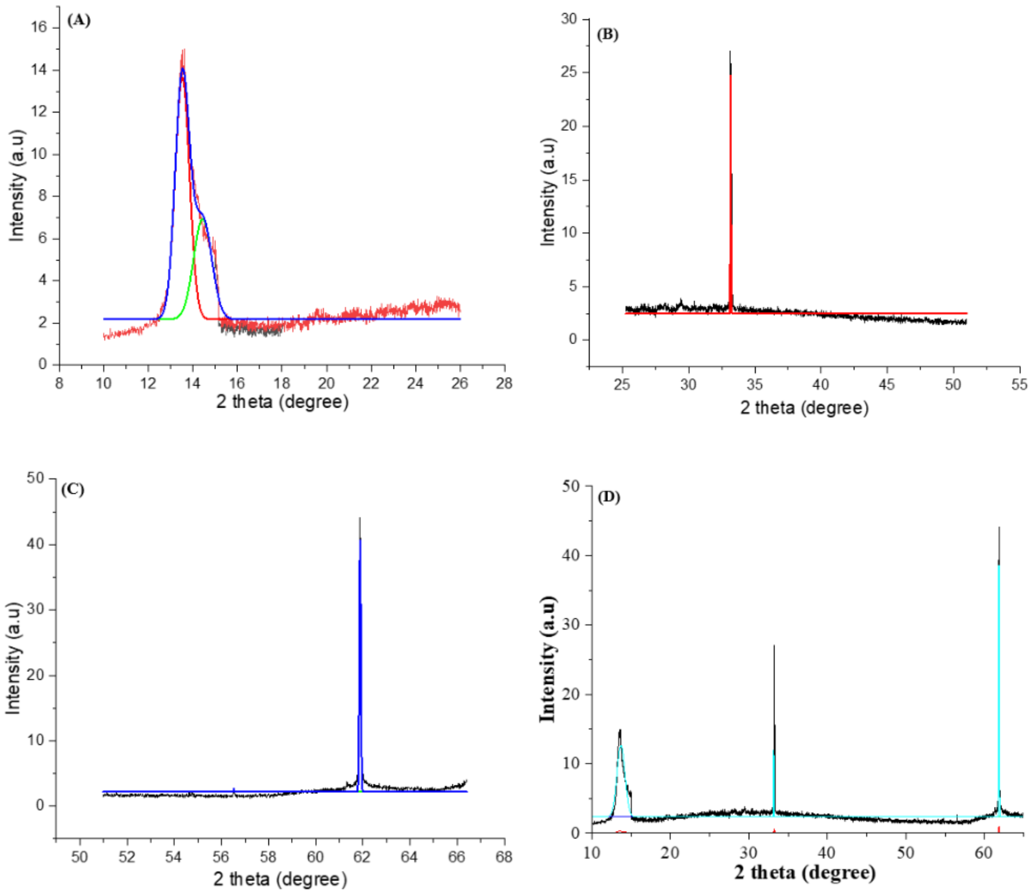

Figure S2. Deconvolution of the Cdots XRD peaks (A, B, and C) was performed using Lorentzian fitting with OriginPro 2023. Panel D shows the deconvolution applied across all XRD diffractograms.

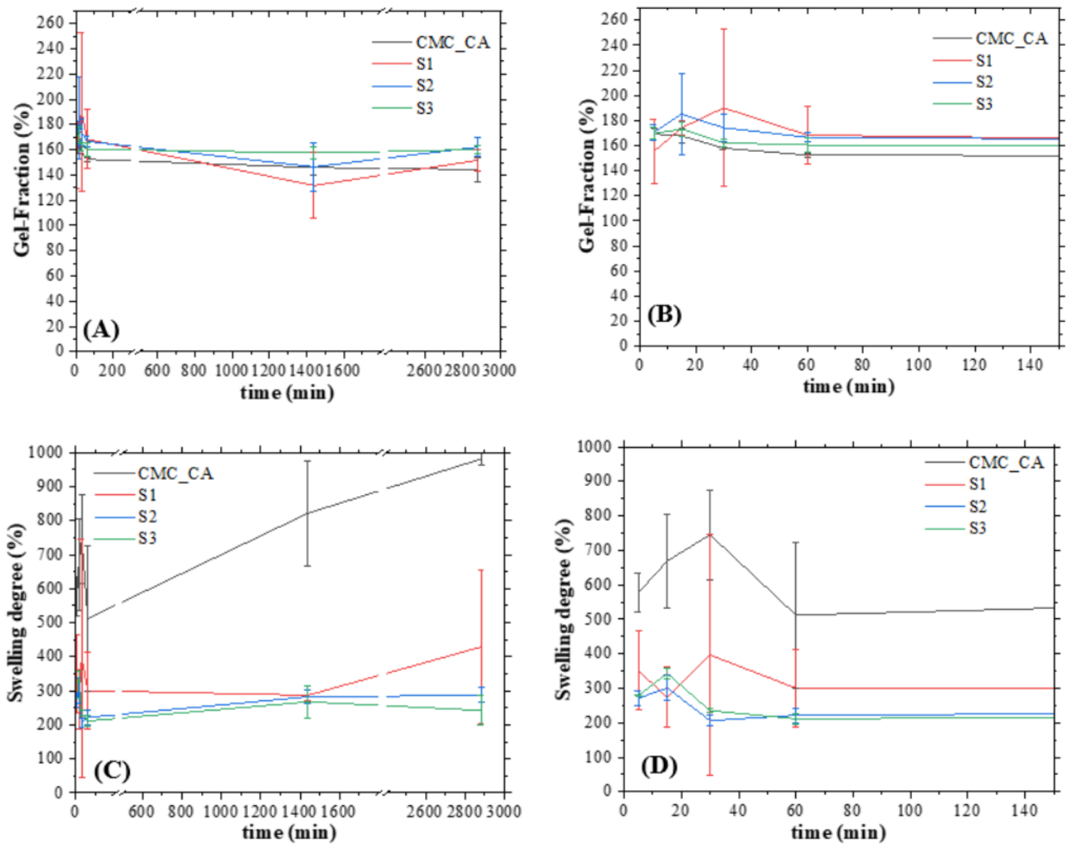

Figure S3. (A) Gel-Fraction of hydrogels in SBF (B) Representing a zoom in the first minutes of Gel-Fraction analysis of hydrogels; (C) Swelling degree of hydrogels in SBF; (D) Representing a zoom in the first minutes of Swelling analysis of hydrogels

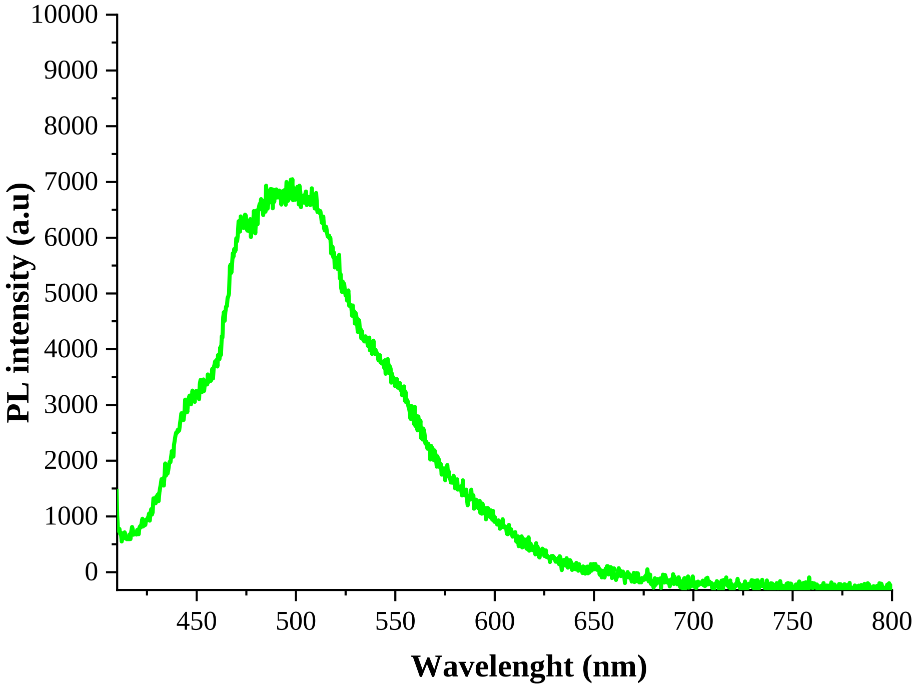

Figure S4. Photoluminescence of CMC at 405 nm, demonstrating that pure CMC exhibits luminescent properties, which may enhance the luminescence of the hydrogel when combined with Cdots.
